# Psychological effects of a low-grade abnormal cervical smear test result: anxiety and associated factors

**DOI:** 10.1038/sj.bjc.6603086

**Published:** 2006-04-04

**Authors:** N M Gray, L Sharp, S C Cotton, L F Masson, J Little, L G Walker, M Avis, Z Philips, I Russell, D Whynes, M Cruickshank, C M Woolley

**Affiliations:** 1 University of Aberdeen and Grampian University Hospitals, Aberdeen, UK; 2 National Cancer Registry Ireland, Cork, Ireland; 3 University of Ottawa, Ontario, Canada; 4 The Postgraduate Medical Institute, University of Hull, in association with Hull York Medical School, UK; 5 Universtiy of Nottingham, Queens Medical Centre & City Hospital, Nottingham, UK; 6 Institute of Medical & Social Care Research, University of Wales Bangor, Bangor, UK

**Keywords:** cervical intraepithelial neoplasia, mass screening, psychological factors, anxiety, questionnaires

## Abstract

Receipt of an abnormal cervical smear result often generates fear and confusion and can have a negative impact on a woman's well-being. Most previous studies have focussed on high-grade abnormal smears. This study describes the psychological and psychosocial effects, on women, of having received a low-grade abnormal smear result. Over 3500 women recruited to TOMBOLA (Trial Of Management of Borderline and Other Low-grade Abnormal smears) participated in this study. Anxiety was assessed using the Hospital Anxiety and Depression Scale (HADS) at recruitment. Socio-demographic and lifestyle factors, locus of control and factors associated with the psychosocial impact of the abnormal smear result were also assessed. Women reported anxiety levels consistent with those found in previous studies of women with high-grade smear results. Women at highest risk of anxiety were younger, had children, were current smokers, or had the highest levels of physical activity. Interventions that focus particularly on women's understanding of smear results and pre-cancer, and/or directly address their fears about cancer, treatment and fertility might provide the greatest opportunity to reduce the adverse psychosocial impact of receiving a low-grade abnormal cervical smear result.

The United Kingdom NHS Cervical Screening Programmes (CSPs) have reduced the incidence of, and mortality from, cervical cancer (Quinn *et al*, 1999; Sasieni and Adams, 1999). However, the screening test has a high degree of sensitivity, resulting, each year, in over 250 000 cervical smears showing abnormalities (NHS Cervical Screening Programmes, 2003; NHS Scotland, Information and Statistics Division, 2004). For women, receipt of an abnormal smear test result frequently leads to heightened levels of anxiety (Bell *et al*, 1995; Gath *et al*, 1995; Maissi *et al*, 2004). Women who have received an abnormal smear result have reported frequent worries and feeling worse about their body (Lerman *et al*, 1991; Wardle *et al*, 1995). Often an abnormal smear result leads to a fear on the part of the woman that she has cancer (Doherty *et al*, 1991; Lerman *et al*, 1991; Somerset and Peters, 1998) and to feelings of self-blame, sexual guilt and concerns about infertility (McDonald *et al*, 1989; Quilliam, 1990; Kavanagh and Broom, 1997).

The overwhelming majority of abnormal smears detected each year are low-grade (i.e. borderline nuclear abnormalities (BNA) or mild dyskaryosis). Most previous research on the psychosocial impact of an abnormal smear has focussed on women with high-grade abnormal smears (i.e. those showing moderate or severe dyskaryosis) and has tended to recruit women attending for a colposcopic examination, making it difficult to separate the psychosocial sequelae of the smear test result itself from the well-documented procedural distress arising from colposcopy (Posner and Vessey, 1988; Marteau *et al*, 1990). It might be expected that psychosocial effects may differ according to the severity of the smear abnormality, and the management strategy adopted. Few studies of the effects of abnormal smear results have included low-grade abnormalities. Moreover, the available studies have been limited in terms of size, by a lack of distinction between different grades of abnormality, or by confounding by management/follow-up (Bell *et al*, 1995; Maissi *et al*, 2004). Thus, the factors associated with adverse psychosocial sequelae among women with low-grade abnormal smears have not been well elucidated.

This paper focuses on the psychological and psychosocial effects associated with receipt of a low-grade abnormal smear test result. Over 3500 women participated in the study, all of whom were recruited into the TOMBOLA trial (Trial Of Management of Borderline and Other Low-grade Abnormal smears), a pragmatic randomised controlled trial of management policies for women with low-grade abnormal smears (Sharp, 2002). The aims of the study were to: (1) quantify the levels of anxiety and depression associated with having received a low-grade abnormal smear result, (2) identify factors associated with increased levels of anxiety, and (3) identify whether the psychosocial impact of the abnormal smear result is higher in particular subgroups of women (for example, defined by age, smear result grade and prior history of another low-grade abnormal smear).

## MATERIALS AND METHODS

### Subjects

A total of 3731 TOMBOLA participants recruited between February 2001 and January 2003 took part in the detailed psychosocial evaluations. Eligible women were aged 20–59 years, had had a smear (termed the index smear) taken routinely as part of the NHS CSPs that showed a low-grade abnormality (either mild dyskaryosis or BNA), had no more than one BNA smear in the previous three years, and were resident in the Grampian Health Board area, Tayside Health Board area or in the Nottingham area. Women were ineligible if they were pregnant at the time of recruitment or had had previous destructive or excisional treatment for proven or suspected cervical lesions. Recruitment to TOMBOLA consisted of sending an information leaflet together with an appointment to attend a hospital-based recruitment clinic to eligible women. Women eligible for psychosocial evaluations, who had provided informed consent, were asked to complete a socio-demographic and lifestyle questionnaire and a baseline psychosocial assessment.

### Materials/measures

The socio-demographic and lifestyle questionnaire collected information including ethnic group, marital status, education since leaving school, employment status, pregnancy and childbirth, smoking habits and physical activity. Information on time from index smear to recruitment was obtained from the trial database. The baseline psychosocial booklet included the Hospital Anxiety and Depression Scale (HADS) (Zigmond and Snaith, 1983), the Multi-dimensional Health Locus of Control Scale (MHLCS) (Wallston *et al*, 1978) and a questionnaire designed specifically for use within TOMBOLA, the Process Outcome Specific Measure (POSM) (Gray *et al*, 2005).

The HADS is a well-validated instrument used to screen for clinically significant depression and anxiety. It is a self-report inventory that consists of 14 items on two subscales, seven items measuring anxiety and seven measuring depression. Each item is scored on a four-point scale from 0 to 3. The items are summed yielding two subscale scores each ranging from 0 to 21. Following established practice, we categorised women's scores to indicate ‘non-cases’ (scores 0–7), ‘possible cases’ (scores 8–10) and ‘probable cases’ (scores of 11 or more) (Fayers and Machin, 2000). Most women (*n*=3530) completed all of the questions on the HADS. For those who had completed at least 50% of either the anxiety or depression subscales (*n*=35 (<1%); *n*=31 (<1%), respectively), best subset regression (StataCorp, 2003) was used to impute scores. The remaining women were excluded from the analyses.

The MHLCS measures three dimensions of health locus of control: ‘internality’; ‘chance externality’; and ‘powerful others externality’ (Wallston *et al*, 1978). The scale consists of three six-item subscales, scored using a six-point forced choice response scale, which ranges from 1 ‘strongly disagree’ to 6 ‘strongly agree’. Possible scores on each subscale range from 6 to 36. As recommended by the MHLCS authors, a score was calculated for respondents who had completed at least four out of six questions for each subscale (Wallston, 2004). For the internal subscale 109 (3%) women had scores imputed. For the powerful others subscale 152 (4%) women had scores imputed and for the chance subscale 170 (5%) women had scores imputed. Women who failed to complete at least four questions on a subscale were excluded from the analysis: *n*=79 (2%) for the internal subscale, *n*=80 (2%) for the powerful others subscale, and *n*=91 (3%) for the chance subscale.

The POSM consists of 16 questions, 11 of which are framed in the form of forced choice personalised statements using a six-point Likert style response format ranging from ‘strongly agree’ to ‘strongly disagree’, and two that relate to change and include a no change response option (Gray *et al*, 2005). There are two filter questions, which allow respondents to skip questions not applicable to them. There is one question that asks about perceived risk of developing cervical cancer in the future. The POSM was included in the analysis to help identify factors likely to be particularly relevant to the management of low-grade abnormalities, which may be associated with raised levels of anxiety. Levels of missing data did not exceed 4% for any one question.

### Analysis

Univariate analysis using the *χ*^2^-test was used to investigate associations between anxiety and socio-demographic factors, depression, POSM and MHLCS. Owing to the very small proportion of women scoring ⩾11 on the HADS depression subscale, all scores of 8 or more on this subscale were combined into one category (possible and probable cases combined). The MHLCS subscale scores were divided into tertiles. The responses to the POSM were combined to produce either a dichotomous outcome (i.e. agree/disagree) or three-point response outcomes (e.g. change for the better/no change/change for the worse).

Factors associated with anxiety were investigated using multiple logistic regression to compute odds ratios (OR) using STATA 8.0 (StataCorp, 2003). The binary outcome variable was <8 and ⩾8. This categorisation was chosen because (1) the aim of interventions to minimise or reduce anxiety would be to render subjects ‘non-cases’ (i.e. to have a score of less than 8 on the anxiety subscale), and (2) the three-point categorisation would not have permitted stable estimates to be obtained from the multivariate analysis. A range of socio-demographic and lifestyle factors, the HADS depression subscale, the three dimensions of the MHLCS and all of the 14 informative questions of the POSM were considered as potential explanatory variables. A multivariate model was developed using a nested approach; if the *P*-value for the likelihood ratio test of the change in deviance (−2 × log likelihood) between a model containing a particular variable, and a model not containing this variable, was less than 0.1, the variable was retained in the model. The goodness-of-fit of each model was checked using the Hosmer & Lemeshow test (Hosmer and Lemeshow, 1989) and the final model reported fits the data adequately (*P*=0.666).

## RESULTS

Of the 3731 women who attended a recruitment appointment and consented to participate in the TOMBOLA psychosocial study, 3671 (98%) completed both a socio-demographic and psychosocial questionnaire.

The mean age of participants was 34 (standard deviation (s.d.)=10.6 years). Forty-two per cent of women were in the 20–29 year age group (Table 1). Twent-four per cent were recruited on the basis of a mild smear and 76% on the basis of a BNA smear. 5% of women had had a BNA smear in the three years before the index smear: 24 of these women had a mild index smear and 155 a smear showing BNA. The median time from index smear to recruitment (and hence completion of the questionnaires) was 71 days. Ninety-six per cent of women described their ethnic group as white. Slightly more than half were married or living as married (56%) and half were in full-time employment (50%). Slightly more women had been to college/university (54%) than had not (47%). Almost half of the women reported that they had never smoked, 35% that they were current smokers and 17% that they were ex-smokers.

Over half of the women (57%) were classed as being a non-case (scored <8 on the HADS anxiety subscale). A fifth of women had scores consistent with being possible cases (scored 8–10) and almost a quarter (23%) had scores that indicated a probable clinically significant level of anxiety (scored ⩾11). The vast majority of women (91%) were classed as non-cases on the HADS depression subscale (scored <8). The mean MHLCS score for the internal subscale was 26 (s.d. 4.3). For the powerful others subscale the mean score was 17 (s.d. 5.9) and for the chance subscale the mean score was 19 (s.d. 5.3).

In univariate analyses, statistically significant associations were found between anxiety and age, trial centre, marital status, employment status, training, physical activity, ever having had a child, and smoking status (Table 2). The associations with age, physical activity, ever having had a child and smoking status remained in the multivariate analysis. A lower proportion of older women (aged 50–59) scored 8–10 or ⩾11 on the HADS anxiety subscale than women in other age groups (*χ*^2^=16.89, *P*=0.010). When those scoring ⩾8 on the HADS anxiety subscale were combined, the multivariate OR for the 50–59 *vs* the 20–29 age groups was statistically significantly less than unity (OR=0.68, 95% confidence interval (CI)=0.48–0.97). Women exercising >3 times per week were more likely to be classed as probable cases (26%) or possible cases (22%) than women who took exercise less than once per week or took no exercise (22% probable cases and 19% possible cases). The multivariate risk estimate for the most active group *vs* the least active was statistically significantly raised (OR=1.52, 95% CI=1.26–1.85). Women who had had children were significantly more likely to be anxious than women who never had children (OR=1.26, 95% CI=1.03–1.55). This was accounted for by an increased proportion scoring ⩾11 among those having had children. 29% of current smokers were classified as probable cases compared to 22% of ex-smokers and 19% of never smokers. The OR for current smokers was significantly higher than unity (OR=1.52, 95% CI=1.26–1.84). There was little evidence of an association between anxiety and either index smear status or previous history of a BNA smear (Table 2). The time between a woman's index smear test and date of completion of the psychosocial questionnaire (recruitment date) was not related to HADS anxiety score (data not shown).

There was a very strong association between the HADS anxiety and the HADS depression scores. Ninety-five per cent of women who scored ⩾8 on the depression subscale also scored ⩾8 on the anxiety subscale (Table 3). The strength of the association was reflected in the OR of 29.14 (95% CI=16.22–52.37). In the univariate analyses, there were significant associations between all three locus of control subscales and anxiety status. However, the association with the chance subscale did not persist in the multivariate analysis. Risk of anxiety decreased with increasing score on the internal subscale (*P* for trend=0.008).

In the univariate analysis there were statistically significant associations between anxiety and all but three of the POSM questions – these questions related to (1) whether the information received answered concerns about the smear result (question 2), (2) future cervical screening intentions (question 13), and (3) belief about regular screening reducing the risk for cervical cancer (question 14). In the multivariate analysis, there were significant associations between anxiety and worries about general health, feelings about self, worries about cervical cancer, future fertility, sex life, perceived risk of cervical cancer and support. Cases of anxiety were more common among women who felt worse about themselves since receiving their smear result (OR=2.07, 95% CI=1.70–2.53). There were very strong positive relations between anxiety and worries that the next smear would show changes to the cells, worries about having cervical cancer, worries about future fertility and worries about having sex. Fifty per cent of women reporting that their sex life had changed for the worse were probable cases compared to 19% reporting no change and 25% reporting change for the better.

## DISCUSSION

We found that 23% of women who had recently received a low-grade abnormal cervical smear test result scored ⩾11 on the anxiety subscale of the HADS, and a further 20% scored between 8 and 10. The frequency scoring ⩾11 was substantially higher than that observed in women in a non-clinical general adult UK population (16%) (Crawford *et al*, 2001). Moreover, our findings are consistent with those from a study of women who had received higher-grade abnormal cervical smear results (Bell *et al*, 1995).

Age was inversely associated with anxiety in our final model (*P*=0.031), although the risk estimate was only statistically significant for women in the oldest age group (50–59 years). It may be that older women had fewer worries about issues such as future fertility and their sex lives and thus, were not as concerned by the smear result. Screening uptake in the UK exceeds 80% (NHS CSP, 2004; ISD Scotland, 2005) and the frequency of low-grade abnormal smears is highest in women under 30 and declines with age (10.5% of smears in this age group, compared to 5.7, 4.7 and 2.8% in the 30–39, 40–49 and 50–59 age groups, respectively) (NHS Health and Social Care Information Centre, 2005). The implication of our results is that considerable numbers of younger women could be experiencing adverse psychosocial consequences of screening. At the time participants were recruited to the study, the NHS CSPs in England and Scotland screened women aged 20–59 (Scotland) or 64 (England). Since then, and in response to analyses suggesting that smears are not as effective in younger women (Sasieni *et al*, 2003), the programme in England has raised the lower screening limit to 25 (http://www.cancerscreening.nhs.uk/cervical). This age limit is consistent with programmes in countries including Norway, Italy, France and Belgium, although is still younger than the lower age limit in some other countries (e.g. Finland, Sweden, Netherlands) (IARC Working Group, 2005). It might be suggested that the levels of anxiety experienced by younger women with low-grade smears provides a further argument for excluding these women from screening. It is noteworthy, however, that in our analysis the risk of having a HADS anxiety score of ⩾8 was essentially the same in women aged 30–39 as those aged 20–29 (multivariate OR 30–39 *vs* 20–29=0.97). Moreover, 26% of the 30–39 age group were classified as definite cases (score ⩾11) compared to 23% of the 20–29 age group. Thus, our observation of an inverse association between age and anxiety is not simply a consequence of higher levels in the youngest women; there are considerable levels of anxiety in women aged 30–39 and this needs to be addressed.

A mild smear corresponds to a higher grade of abnormality than a BNA smear; however, there was no relation between smear status and HADS anxiety score. There are at least two possible explanations for this. First, before their TOMBOLA recruitment appointment, women may not have been told explicitly what their smear result was. We are aware that women may not be told the grade of their smear result, but simply that it is abnormal. This is supported by a study from a single health authority (Nottingham), which showed that there was considerable variation in both the method and content of communications delivering mild and BNA smear results (Philips *et al*, 2002). Second, women may have been told the grade of the smear but may not have understood its clinical significance. For example, a BNA smear may have been interpreted as being ‘borderline cancer’ rather than ‘borderline normal’. It appears that women often do not understand the purpose and indications of the cervical smear (Fylan, 1998), or the meaning of pre-cancer, and erroneously conclude that any abnormalities detected by screening must indicate cancer (Kavanagh and Broom, 1997). As our results indicate that there are similar levels of anxiety overall among women with low-grade smears as among women with high-grade smears, it seems likely that it is the receipt of an abnormal smear result *per se*, irrespective of the grade, which engenders adverse psychosocial consequences. We anticipated that women who had had a BNA smear result in the three years before their index smear would be more anxious than women who had not previously had an abnormal smear. However, there was no relationship between prior BNA smear and anxiety. In part this may have been due to the relatively small numbers of women in our study who had had a prior BNA smear (*n*=175, 5% overall). Alternatively, it is possible that receipt of an abnormal smear causes a spike in anxiety that resolves over time, thus a smear taken up to 3 years ago might not impact on current anxiety levels. A recent study showed that whereas informing women that they had an abnormal smear (with or without HPV testing) was associated with raised levels of state anxiety and general distress in the first month following receipt of results, this was no longer evident 6 months later (Maissi *et al*, 2005).

Elucidation of factors associated with raised levels of anxiety is likely to be helpful in identifying: (1) particularly vulnerable subgroups of the population, (2) particular issues that may be causing concern or worries among women, and (3) targets that could be addressed in interventions aimed at helping alleviate anxiety. Having had children was a significant predictor of scoring ⩾8 on the HADS anxiety subscale. Previous studies have shown that there are higher rates of psychiatric disorders in women with children, although the authors of these studies have suggested that the difference is due to an effect of marriage rather than parity (Dean and White, 1996). In our study, marital status was significantly associated with having had children; over 70% of women who were married/living as married (or who had previously been married) had had children compared to only 19% of single women. Marital status, however, was not associated with anxiety in our multivariate model.

Women who reported themselves to be current smokers were significantly more likely to be anxious than women who had never smoked. Other studies have found an association between smoking and increased anxiety, and poor psychological health has been shown to increase cigarette consumption (Graham and Der, 1999; Bonnet *et al*, 2005). One possible explanation for our finding could be that smokers believe that smoking raises their cervical cancer risk. In our study, women who thought that their chances of getting cancer were higher than average were at increased risk of being anxious and smoking status was significantly associated with belief about chance of getting cancer. Twenty per cent of current smokers believed their chance was higher than average, 74% that it was average and 7% that it was less than average; the comparable figures for never smokers were 18, 71 and 12%.

We found that women who were most physically active were significantly more likely to score ⩾8 on the HADS anxiety subscale. An explanation for this intriguing finding is not obvious, and we might have expected the opposite relation given the positive effects of exercise on endorphins, and hence on mental health generally (Daley, 2002). Although the difficulties in accurate assessment of physical activity levels in epidemiological studies are well known (IARC Working Group, 2002), there is no evidence that reporting varies by level of anxiety. It is possible that women who engage in the highest levels of physical activity are also the most health conscious and, as a result, are most anxious when they receive an abnormal smear result.

Women who were worried about their general health, that their next smear would be abnormal or about having cervical cancer were all at a significantly increased risk of being anxious compared to women who were not worried about these issues. This is consistent with other studies in which receipt of an abnormal smear is associated with women's fears that they have cancer (Doherty *et al*, 1991; Lerman *et al*, 1991; Somerset and Peters, 1998). Women who perceived their chances of developing cervical cancer in the future to be higher than average were at a significantly increased risk of being anxious than women who perceived their chances as average or below average. This is congruent with a recent study of women who had received inadequate smear results among whom perceived risk was found to be a risk factor for state anxiety (French *et al*, 2004). In the current study, anxiety was significantly higher in women who were worried about their ability to have children in the future and in those who had decided to delay getting pregnant. Previous studies have confirmed that women who have had an abnormal smear are concerned about their future fertility and that this may be related to fear about what further treatment might be involved (McDonald *et al*, 1989; Quilliam, 1990).

Two of the POSM questions asked about change in self-perception and change in sex life since receipt of the smear test result. Interestingly, women who indicated any change, whether for the better or for the worse, were more likely to be anxious than women who responded that there had been no change. It may be that the change (for better or for worse) resulted from increased anxiety caused by the abnormal smear result.

Although most of the responses to the POSM questions were associated with anxiety in multivariate analyses, five were not. Two of these questions related to intention to continue having regular smears and belief that regular smears reduce risk of developing cervical cancer. For both of these questions, only small proportions of women (<1 and 3%, respectively) disagreed with the statements. It might have been expected, given the reasonably high overall levels of anxiety in the study population, that women might have felt somehow dissatisfied with the information they had received about their smear result. In contrast, over 90% of women felt well informed about what their smear result meant and 92% were satisfied that the information received had addressed any concerns. This apparent contradiction may suggest that the information that women receive (or source for themselves), and/or the way in which the information is conveyed, is not providing adequate reassurance. Previous research has shown that the way in which smear results are conveyed and the content of the communication varies, not only across, but also within health authorities (Philips *et al*, 2002). Moreover, there is no guarantee that women will understand the information contained in the communication of the smear result. Further study of what information women receive and how different methods of communicating results to women influence levels of anxiety would be useful.

We observed a significant inverse relationship between internal locus of control and anxiety and, compatible with this, women who scored highly on the powerful others externality subscale were at increased risk of anxiety. Other studies have found that an external locus of control is significantly associated with anxiety disorders and that women who score highly on the internal subscale are more likely to report good health (Raja *et al*, 1994; Beekman *et al*, 2000). In relation to communication and information provision, a recent study found that matching health messages to an individual's health locus of control in women resulted in higher attendance for a mammogram (Williams-Piehota *et al*, 2004). It may be possible to assess locus of control in the context of cervical screening and to present information that is targeted to match the individual's locus of control, for example, when women access web-based health information.

We assessed anxiety using the HADS, a screening (rather than diagnostic) test for clinically significant anxiety (and depression) which is widely used in both clinical and non-clinical settings (Crawford *et al*, 2001; Strik *et al*, 2004). Although we were not in a position to cross validate the HADS cut-offs for possible and probable cases with a standardised diagnostic interview, in pilot testing the HADS proved reliable in women recruited to TOMBOLA (Gray *et al*, 2005). In terms of classifying respondents, investigators have used a variety of schemes, including cut-offs at 8 (Osborne *et al*, 2003) and 11 (Pascoe *et al*, 2000). We decided to take a score of ⩾8 as indicating a level of anxiety that could be considered abnormal and, therefore, may warrant intervention. The main reason for choosing 8 as a cut-off was that we wanted to ensure that any suggested interventions arising from the analyses would be relevant to the greatest number of women (i.e. not just to women scoring above 10). The aim of such interventions would be to render the maximum number of women ‘non-cases’ (i.e. to have a score of less than 8 on the anxiety subscale); thus, it would not be sufficient to move women who were probable cases to possible cases. A secondary consideration was that we wanted the estimates from our multivariate analyses to be stable making sure that we had an adequate number of subjects in each analysis cell/subgroup: using a cut-off of 8, rather than 11, helped to ensure this. We undertook a sensitivity analysis to determine whether our results were dependent on the chosen cut-point. The analysis was repeated using a score of 11 or more to identify ‘cases’ with those scoring 10 or less classified as ‘non-cases’. The results were essentially unchanged; the only substantive difference was that level of post-school education entered the model as a significant predictor of anxiety.

### Strengths and limitations of the study

Our study is one of the very few to have focussed only on women with low-grade abnormal smears. As far as we are aware, it is the largest study of the psychological status of women with such abnormalities and, including more than 3500 women, it is one of the largest studies of the determinants of anxiety to have been reported.

Participants were recruited from the cervical screening programmes in Scotland and England and are likely to be representative of the UK screening population as a whole. The three study centres comprise both rural and city-based populations and incorporate affluent and deprived areas and the centre in England was ethnically diverse. Ninety-eight per cent of women asked to complete the psychosocial questionnaires did so. Furthermore, we measured anxiety prior to women knowing how they would be followed-up so there is no contamination of the results with any effects of treatment. Women in TOMBOLA are being followed for 3 years, over which time they will complete psychosocial questionnaires at four time points. This will permit temporal trends in anxiety to be charted.

Women were asked to complete the questionnaires at a recruitment appointment in a hospital-based clinic setting and it is possible that attending the appointment contributed to anxiety. Maissi *et al*, 2004 found that whether an abnormal smear is a woman's first smear may be a predictor of concern about the test result. Our access to a woman's smear history before her participation in TOMBOLA was restricted. Therefore, we could not identify whether the abnormal smear that made a woman eligible for inclusion in TOMBOLA was her first cervical smear. However, the lack of a substantial difference in anxiety between women aged 20–29 and women aged 30–39 suggests that our results are not driven by the effect of first smears. Twenty-one per cent of women aged 20–29 scored between 8 and 10 compared to 20% of women aged 30–39; furthermore, 23% of women aged 20–29 scored ⩾11 compared to 26% of women aged 30–39. Although standardised trial information leaflets were given to all participants, women received their smear results in the usual way and this differed between and within centres. We also had no control over the information women received from other health professionals or sourced for themselves. This undoubtedly led to variation in the amount and quality of information women received and also in the means of communication.

### Implications and conclusions

Assessing the levels and determinants of psychological and psychosocial consequences of receiving a low-grade abnormal smear result is of considerable public health importance. As low-grade smears account for the majority of cervical smears classed as abnormal in the UK, the results of our study suggest that significant numbers of women could be incurring adverse psychological and psychosocial consequences of screening. We have found a high prevalence of anxiety among women who have a low-grade smear, and that the proportion scoring in the abnormal range is consistent with previous studies of women with high-grade smear results. We further found that those who are at highest risk of anxiety tend to be younger, have children, be current smokers, or have higher levels of physical activity. These may represent particularly vulnerable subgroups of the screening population. We will further investigate the determinants of anxiety in the analysis of the TOMBOLA longitudinal data. Strategies are needed to minimise the adverse effects of a low-grade smear result on women. Interventions that focus particularly on women's understanding of smear results and pre-cancer, and those that directly address their fears about cancer, treatment and fertility might provide greatest opportunity to reduce the adverse psychosocial impact of receiving a low-grade abnormal cervical smear result.

## Figures and Tables

**Table 1:** Socio-demographic characteristics of respondents participating in the baseline psychological assessment within TOMBOLA

	***n***	**%**
*Age group*		
20–29 years	1551	42
30–39 years	982	27
40–49 years	797	22
50–59 years	341	9
		
*Index smear status*		
Mild	882	24
BNA	2789	76
		
*Previous smear history in the 3 years before index smear*		
No abnormal smear	3492	95
One BNA	179	5
		
*Trial centre*		
A	1207	33
B	882	24
C	1582	43
		
*Ethnic group*		
White	3515	96
Non-white	148	4
Missing	8	—
		
*Marital status*		
Married/living as married	2042	56
Divorced/separated/widowed	492	14
Single	1107	30
Missing	30	—
		
*Employment status*		
Full-time paid employment	1819	50
Part-time paid employment	864	24
Student	340	9
Not in paid employment	645	18
Missing	3	—
		
*Training*		
None	990	27
Through work with qualification	725	20
Qualification other than degree from college/university	1046	29
Degree from college/university	901	25
Missing	9	—
		
*Physical activity*		
<Once/week	1456	40
1–3 times/week	867	24
>3 times/week	1310	36
Missing	38	—
		
*Ever had children*		
Yes	2048	56
No	1591	44
Missing	32	—
		
*Smoking status*		
Never smoker	1760	48
Ex-smoker	627	17
Current smoker	1260	35
Missing	24	—

**Table 2:** Associations between the HADS anxiety subscale and socio-demographic and lifestyle factors

**Hospital Anxiety and Depression Scale – anxiety subscale**								
	**Non-case (<8) (*n*=2033)**		**Doubtful case (8–10) (*n*=711)**		**Probable case (>10) (*n*=818)**		**Multivariate analysis^a^**	
	***n***	**%**	***n***	**%**	***n***	**%**	**OR^b^**	**(95% CI)**
*Age group*								
20–29 years	850	57	308	21	341	23	1.00	(ref)
30–39 years	516	54	189	20	253	26	0.97	0.78–1.22
40–49 years	456	59	154	20	169	22	0.85	0.66–1.10
50–59 years	211	65	60	18	55	17	0.68	0.48–0.97
*P*-value from *χ*^2^-test						**0.010**		
*P*-value from *χ*^2^-test for trend							**0.031**	
Global *P*-value							0.133	
								
*Index smear status*								
Mild	477	56	161	19	213	25	1.00	(ref)
BNA	1556	57	550	20	605	22	1.09	0.90–1.32
*P*-value from *χ*^2^-test						0.236		
Global *P*-value							0.397	
								
*Previous smear history in the 3 years before index smear*								
No abnormal smear	1930	57	675	20	782	23	1.00	(ref)
One BNA	103	59	36	21	36	21	0.93	0.64–1.37
*P*-value from *χ*^2^-test						0.742		
Global *P*-value							0.722	
								
Median time from index smear to recruitment (days)	72		70		70			
								
*Trial centre*								
A	707	61	237	20	214	18	1.00	(ref)
B	470	54	169	19	231	27	1.06	0.85–1.33
C	856	56	305	20	373	24	0.95	0.79–1.16
*P*-value from *χ*^2^-test						**<0.001**		
Global *P*-value from LR test							0.595	
								
*Ethnic group*								
White	1955	57	671	20	788	23	1.00	(ref)
Non-white	78	56	35	25	27	19	0.80	0.51–1.25
*P*-value from *χ*^2^-test						0.242		
Global *P*-value from LR test							0.324	
								
*Marital status*								
Married/living as married	1155	58	368	19	464	23	1.00	(ref)
Divorced/separated/widowed	251	53	99	21	124	26	0.98	0.76–1.28
Single	610	57	240	22	222	21	0.90	0.71–1.13
*P*-value from *χ*^2^-test						**0.019**		
Global *P*-value from LR test							0.638	
								
*Employment status*								
Full-time paid employment	1075	60	349	20	355	20	1.00	(ref)
Part-time paid employment	483	57	172	20	186	22	0.97	0.77–1.21
Student	175	55	72	23	72	23	1.04	0.77–1.40
Not in paid employment	298	48	118	19	204	33	1.02	0.79–1.33
*P*-value from *χ*^2^-test						**<0.001**		
Global *P*-value from LR test							0.970	
								
*Training*								
None	522	54	180	19	258	27	1.00	(ref)
Through work with qualification	378	54	150	21	176	25	1.14	0.89–1.45
Qualification other than degree from college/university	616	60	190	19	214	21	0.84	0.67–1.06
Degree from college/university	512	59	190	22	167	19	0.97	0.75–1.25
*P*-value from *χ*^2^-test						**0.001**		
Global *P*-value from LR test							0.096	
								
*Physical activity*								
<Once/week	838	60	263	19	304	22	1.00	(ref)
1–3 times/week	509	60	159	19	181	21	1.13	0.91–1.40
>3 times/week	663	52	283	22	324	26	1.52	1.26–1.85
*P*-value from *χ*^2^-test						**0.001**		
*P*-value from *χ*^2^-test for trend							**<0.001**	
Global *P*-value from LR test							**<0.001**	
								
*Ever had children*								
No	931	60	307	20	311	20	1.00	(ref)
Yes	1085	55	401	20	497	25	1.26	1.03–1.55
*P*-value from *χ*^2^-test						**0.001**		
Global *P*-value from LR test							**0.025**	
								
*Smoking status*								
Never smoker	1070	63	320	19	319	19	1.00	(ref)
Ex-smoker	350	58	123	20	132	22	1.22	0.97–1.54
Current smoker	603	49	262	21	360	29	1.52	1.26–1.84
*P*-value from *χ*^2^-test						**<0.001**		
Global *P*-value from LR test							**<0.001**	

a Outcome either 0=non-cases (<8) or 1=definite or doubtful cases (=>8).

b Mutually adjusted for: ever had children, training, smoking status, physical activity, age, depression, internal subscale, powerful others subscale, POSM q3–6, q10, q11, q15, q16. 139 women did not complete a sufficient number of questions on one or more of the outcome measures and were excluded from the analysis.

Bold values indicate statistically significant results.

**Table 3:** Associations between the HADS anxiety subscale and the HADS depression subscale status, baseline MHLCS status and POSM responses

**Hospital Anxiety and Depression Scale – anxiety subscale**								
	**Non-case (<8) (*n*=2033)**		**Possible case (8–10) (*n*=711)**		**Case (>10) (*n*=818)**		**Multivariate analysis^a^**	
	***n***	**%**	***n***	**%**	***n***	**%**	**OR^b^**	**(95% CI)**
*Depression subscale*								
Non-case (<8)	2017	62	659	20	566	17	1.00	(ref)
Doubtful/probable case (8 or more)	15	5	51	16	251	79	29.14	16.22–52.37
*P*-value from *χ*^2^-test						**<0.001**		
Global *P*-value from LR test							**<0.001**	
								
*MHLCS internal (tertiles)*								
Low	580	53	225	20	294	27	1.00	(ref)
Medium	592	58	203	20	221	22	0.87	0.70–1.07
High	834	59	278	20	295	21	0.76	0.63–0.93
*P*-value from *χ*^2^-test						**0.004**		
*P*-value from *χ*^2^-test for trend							**0.008**	
Global *P*-value from LR test							**0.030**	
								
*MHLCS chance (tertiles)*								
Low	697	62	195	17	234	21	1.00	(ref)
Medium	557	58	196	20	213	22	0.99	0.80–1.23
High	744	52	315	22	360	25	1.08	0.88–1.33
*P*-value from *χ*^2^-test						**<0.001**		
*P*-value from *χ*^2^-test for trend							0.430	
Global *P*-value from LR test							0.627	
								
*MHLCS powerful others (tertiles)*								
Low	668	65	178	17	178	17	1.00	(ref)
Medium	719	60	203	17	267	22	0.96	0.78–1.19
High	618	47	325	25	365	28	1.55	1.26–1.91
*P*-value from *χ*^2^-test						**<0.001**		
*P*-value from *χ*^2^-test for trend							**<0.001**	
Global *P*-value from LR test							**<0.001**	
								
POSM questions								
*1 In general I feel well enough informed about what my smear result means.*								
Agree	1835	58	630	20	702	22	1.00	(ref)
Disagree	184	50	74	20	112	30	1.22	0.93–1.60
*P*-value from *χ*^2^-test						**0.001**		
Global *P*-value from LR test							0.151	
								
*2 The information I have received has answered the concerns I have had about my smear result.*								
Agree	1845	57	649	20	731	23	1.00	(ref)
Disagree	113	50	47	21	65	29	1.24	0.89–1.73
*P*-value from *χ*^2^-test						0.067		
Global *P*-value from LR test							0.208	
								
*3 Since getting my smear result I have been worried about my general health.*								
Disagree	820	77	126	12	118	11	1.00	(ref)
Agree	1196	48	577	23	697	28	1.83	1.47–2.28
*P*-value from *χ*^2^-test						**<0.001**		
Global *P*-value from LR test							**<0.001**	
								
*4 Since getting my smear result the way I feel about myself has changed.*								
Neither better nor worse	1547	67	388	17	370	16	1.00	(ref)
For the better	140	43	80	25	105	32	1.82	1.37–2.43
For the worse	322	36	231	26	335	38	2.07	1.70–2.53
*P*-value from *χ*^2^-test						**<0.001**		
Global *P*-value from LR test							**<0.001**	
								
*5 Since getting my smear result I have been worried that my next smear will show changes to the cells.*								
Disagree	307	80	46	12	29	8	1.00	(ref)
Agree	1707	54	658	21	783	25	1.48	1.03–2.12
*P*-value from *χ*^2^-test						**<0.001**		
Global *P*-value from LR test							**0.031**	
								
*6 Since getting my smear result I have been worried that I may have cervical cancer.*								
Disagree	846	75	167	15	121	11	1.00	(ref)
Agree	1165	49	536	22	691	29	1.50	1.22–1.85
*P*-value from *χ*^2^-test						**<0.001**		
Global *P*-value from LR test							**<0.001**	
*8 Since getting my smear result I have been worried about my ability to have children in the future.* c								
Disagree	359	71	84	17	66	13	1.00	(ref)
Agree	362	46	183	23	246	31	1.61	1.19–2.18
*P*-value from *χ*^2^-test						**<0.001**		
Global *P*-value from LR test							**0.002**	
								
*9 Because of the follow-up for my smear I have decided to delay getting pregnant.* c								
Disagree	313	61	104	20	94	18	1.00	(ref)
Agree	85	39	49	23	83	38	1.41	0.94–2.12
*P*-value from *χ*^2^-test						**<0.001**		
Global *P*-value from LR test							0.101	
								
*10 Since getting my smear result I have been worried about having sex.*								
Disagree	1623	65	463	18	429	17	1.00	(ref)
Agree	348	37	227	24	366	39	1.75	1.44–2.11
*P*-value from *χ*^2^-test						**<0.001**		
Global *P*-value from LR test							**<0.001**	
								
*12 Since getting my smear result my sex life has changed.* d								
Neither better nor worse	1628	62	500	19	504	19	1.00	(ref)
For the better	29	43	21	31	17	25	1.38	0.76–2.53
For the worse	78	28	61	22	141	50	1.50	1.06–2.13
*P*-value from *χ*^2^-test						**<0.001**		
Global *P*-value from LR test							**0.046**	
								
*13 I intend to continue having regular smears.*								
Agree	1998	57	704	20	809	23	1.00	(ref)
Disagree	8	73	1	9	2	18	1.28	0.31–5.26
*P*-value from *χ*^2^-test						0.535		
Global *P*-value from LR test							0.733	
								
*14 I believe that having regular smears reduces my risk of getting cervical cancer.*								
Agree	1896	57	680	20	775	23	1.00	(ref)
Disagree	72	62	20	17	25	21	0.86	0.54–1.37
*P*-value from *χ*^2^-test						0.545		
Global *P*-value from LR test							0.516	
								
*15 What do think your chances are of developing cervical cancer in the future?*								
Average	1520	60	478	19	526	21	1.00	(ref)
Lower than average	210	61	69	20	63	18	0.99	0.74–1.34
Higher than average	262	41	154	24	217	34	1.71	1.38–2.12
*P*-value from *χ*^2^-test						**<0.001**		
Global *P*-value from LR test							**<0.001**	
								
*16 Since getting my smear result I have generally been satisfied with the support I have had from other people.*								
Agree	1827	58	638	20	705	22	1.00	(ref)
Disagree	130	44	61	21	103	35	1.56	1.15–2.11
*P*-value from *χ*^2^-test						**<0.001**		
Global *P*-value from LR test							**0.004**	

a Outcome either 0=non cases (<8) or 1=definite or doubtful cases (=>8)

b Mutually adjusted for: ever had children, training, smoking status, physical activity, age, depression, internal subscale, powerful others subscale, POSM q3–6, q10, q11, q15, q16. 139 women did not complete a sufficient number of questions on one or more of the outcome measures and were excluded from the analysis.

c In women who did not answer ‘no’ to POSM q7 (Before you received your smear result were you planning to have a child in the future?).

d In women who did not answer ‘no’ to POSM q11 (Are you sexually active?).

Bold values indicate statistically significant results.

## References

[bib1] Beekman ATF, de Beurs E, van Balkom AJLM, Deeg DJH, van Dyck R, van Tilbug W (2000) Anxiety and depression in later life: co-occurrence and communality of risk factors. Am J Psychiatry 157: 89–95 10618018 10.1176/ajp.157.1.89

[bib2] Bell S, Porter M, Kitchener H, Fraser C, Fisher P, Mann E (1995) Psychological response to cervical screening. Prev Med 24: 610–616 8610085 10.1006/pmed.1995.1096

[bib3] Bonnet F, Irving K, Terra J, Nony P, Berthezene F, Moulin P (2005) Anxiety and depression are associated with unhealthy lifestyle in patients at risk of cardiovascular disease. Atherosclerosis 178: 339–344 15694943 10.1016/j.atherosclerosis.2004.08.035

[bib4] Crawford JR, Henry JD, Crombie C, Taylor EP (2001) Normative data for the HADS from a large non-clinical sample. Br J Clin Psychol 40: 429–434 11760618 10.1348/014466501163904

[bib5] Daley AJ (2002) Exercise therapy and mental health in clinical populations: is exercise therapy a worthwhile intervention? Adv Psyhciatr Treat 8: 262–270

[bib6] Dean C, White AP (1996) A twin study examining the effect of parity on the prevalence of psychiatric disorder. J Affect Disord 38: 145–152 8791183 10.1016/0165-0327(96)00006-7

[bib7] Doherty IE, Richardson PH, Wolfe CD, Raju KS (1991) The assessment of the psychological effects of an abnormal cervical smear result and subsequent medical procedures. J Psychosom Obstet Gynecol 12: 319–324

[bib8] Fayers P, Machin D (2000) Quality of Life. Assessment, Analysis and Interpretation. Chichester: John Wiley and Sons Ltd

[bib9] French DP, Maissi E, Marteau TM (2004) Psychological costs of inadequate cervical smear test results. Br J Cancer 91: 1887–1892 15534608 10.1038/sj.bjc.6602224 PMC2409776

[bib10] Fylan F (1998) Screening for cervical cancer: a review of women’s attitudes, knowledge, and behaviour. Br J Gen Prac 48: 1509–1514 PMC1313202 10024713

[bib11] Gath DH, Hallam N, Mynors-Wallis L, Day A, Bond SAK (1995) Emotional reactions in women attending a UK colposcopy clinic. J Epidemiol Community Health 49: 79–83 7707011 10.1136/jech.49.1.79 PMC1060079

[bib12] Graham H, Der G (1999) Patterns and predictors of tobacco consumption among women. Health Educ Res 14: 611–618 10510069 10.1093/her/14.5.611

[bib13] Gray NM, Sharp L, Cotton SC, Avis M, Phillips Z, Russell I, Walker LG, Whynes D, Little J (2005) Developing a questionnaire to measure the psychosocial impact of an abnormal cervical smear result and its subsequent management: the TOMBOLA (Trial Of Management of Borderline and Other Low-grade Abnormal smears) trial. Qual Life Res 14: 1153–1562 10.1007/s11136-004-8146-5 16110935

[bib14] Hosmer DW, Lemeshow S (1989) Applied Logistic Regression. New York: Wiley

[bib15] IARC Working Group (2002) IARC Handbooks of Cancer Prevention Vol. 6: The Role of Weight Control and Physical Activity in Cancer Prevention. Lyon: IARC

[bib16] IARC Working Group (2005) Cervical Cancer Screening: IARC Handbooks of Cancer Prevention. Lyon: IARC

[bib17] Kavanagh AM, Broom DH (1997) Women’s understanding of abnormal cervical smear test results: a qualitative interview study. BMJ 314: 1388–1391 9161314 10.1136/bmj.314.7091.1388 PMC2126646

[bib18] Lerman C, Miller S M, Scarborough R, Hanjani P, Nolte S, Smith D (1991) Adverse psychologic consequences of positive cytologic cervical screening. Am J Obstet Gynecol 165: 658–662 1892194 10.1016/0002-9378(91)90304-a

[bib19] Maissi E, Marteau TM, Hankins M, Moss S, Legood R, Gray A (2004) Psychological impact of human papillomavirus testing in women with borderline or mildly dyskaryotic cervical smear test results: cross sectional questionnaire study. BMJ 328: 1293–1300 15166066 10.1136/bmj.328.7451.1293 PMC420171

[bib20] Maissi E, Marteau TM, Hankins M, Moss S, Legood R, Gray A (2005) The psychological impact of human paillomavirus testing in women with borderline or mildly dyskaryotic cervical smear test results: 6-month follow-up. Br J Cancer 92: 990–994 15785734 10.1038/sj.bjc.6602411 PMC2361952

[bib21] Marteau TM, Walker P, Giles J, Smail M (1990) Anxieties in women undergoing colposcopy. Br J Obstet Gynaecol 97: 859–861 2242376 10.1111/j.1471-0528.1990.tb02586.x

[bib22] McDonald TW, Neutens JJ, Fischer LM, Jessee D (1989) Impact of cervical intraepithelial neoplasia diagnosis and treatment on self-esteem and body image. Gynecol Oncol 34: 345–349 2767527 10.1016/0090-8258(89)90170-4

[bib23] NHS Cervical Screening Programmes (2003) Celebrating 15 years of achievement. NHS Cervical Screening Programme. Annual Review 2003. Sheffield: NHS Cervical Screening Programme

[bib24] NHS Cervical Screening Programmes (2004) Making a difference: NHS Cervical Screening Programme Annual Review 2004. Annual Review 2004. Sheffield: NHS Cervical Screening Programme

[bib25] NHS Health and Social Care Information Centre (2005) Cervical Screening Programme, England: 2004–2005. Bulletin 2005/09/HSCIC, Health Services Community Statistics, England (available at http://www.ic.nhs.uk/pubs/cervicscrneng2005/sb0509.pdf/file)

[bib26] NHS Scotland, Information & Statistics Division (2004) Cervical Cytology Workload Statistics (Quarter ending 30 September 2003). Health Briefing number 04/02. Edinburgh: Information & Statistics Division

[bib27] NHS Scotland, Information & Statistics Division (2005) Cervical Screening Uptake by NHS Board. Edinburgh: Information & Statistics Division.(Available at http://www.isdscotland.org/isd/info3.jsp?pContentID=2388&p_applic=CCC&p_service=Content.show)

[bib28] Osborne RH, Elsworth GR, Hopper JL (2003) Age-specific norms and determinants of anxiety and depression in 731 women with breast cancer recruited through a population-based cancer registry. Eur J Cancer 39: 755–762 12651200 10.1016/s0959-8049(02)00814-6

[bib29] Pascoe S, Edelman S, Kidman A (2000) Prevalence of psychological distress and use of support services by cancer patients at Sydney hospitals. Aust NZ J Psychiatry 34: 785–792 10.1080/j.1440-1614.2000.00817.x 11037364

[bib30] Philips Z, Johnson S, Avis M, Whynes DK (2002) Communicating mild and borderline abnormal cervical smear results: how and what women are told? Cytopathology 13: 355–363 12485171 10.1046/j.1365-2303.2002.00447.x

[bib31] Posner T, Vessey M (1988) Prevention of Cervical Cancer: The Patient’s View. London: King Edward’s Hospital Fund for London

[bib32] Quilliam S (1990) Positive smear. The emotional issues and what can be done. Health Educ J 49: 19–20

[bib33] Quinn M, Babb P, Jones J, Allen E (1999) Effect of screening on incidence of and mortality from cancer of cervix in England: evaluation based on routinely collected statistics. BMJ 318: 904–908 10102852 10.1136/bmj.318.7188.904 PMC27810

[bib34] Raja SN, Williams S, McGee R (1994) Multidimensional health locus of control beliefs and psychological health for a sample of mothers. Soc Soc Med 39: 213–220 10.1016/0277-9536(94)90330-1 8066500

[bib35] Sasieni P, Adams J (1999) Effect of screening on cervical cancer mortality in England and Wales: analysis of trends with an age period cohort model. BMJ 318: 1244–1245 10231253 10.1136/bmj.318.7193.1244 PMC27862

[bib36] Sasieni P, Adams J, Cuzick J (2003) Benefit of cervical screening at different ages: evidence from the UK audit of screening histories. Br J Cancer 89: 88–93 12838306 10.1038/sj.bjc.6600974 PMC2394236

[bib37] Sharp L (2002) How should women with an abnormal cervical smear be followed-up? Scottish Cancer Therapy Network Newsletter 35: 15–17, (Available at http://www.isdscotland.org/isd/files/sctn_news_35.pdf)

[bib38] Somerset M, Peters TJ (1998) Intervening to reduce anxiety for women with mild dyskaryosis: do we know what works and why? J Adv Nurs 28: 563–579 9756224 10.1046/j.1365-2648.1998.00717.x

[bib39] StataCorp (2003) Stata Statistical Software: Release 8.0. College Station. Texas: Stata Corporation

[bib40] Strik JJMH, Lousberg R, Cheriex EC, Honig A (2004) One-year cumulative incidence of depression following myocardial infarction and impact on cardiac outcome. J Psychosom Res 56: 59–66 14987965 10.1016/S0022-3999(03)00380-5

[bib41] Wallston KA (2004) Multidimensional Health Locus of Control (MHLC) scales(Available at http://www.vanderbilt.edu/nursing/kwallston/mhlcscales.htm) 10.1177/109019817800600107 689890

[bib42] Wallston KA, Wallston BS, DeVellis R (1978) Development of the multidimensional health locus of control (MHLC) scales. Health Educ Monogr 6: 160–170 689890 10.1177/109019817800600107

[bib43] Wardle J, Pernet A, Stephens D (1995) Psychological consequences of positive results in cervical-cancer screening. Psychol Health 10: 185–194

[bib44] Williams-Piehota P, Schneider TR, Pizarro J, Mowad L, Salovey P (2004) Matching health messages to health locus of control beliefs for promoting mammography utilization. Psychol Health 19: 407–423

[bib45] Zigmond AS, Snaith RP (1983) The hospital anxiety and depression scale. Acta Psychiatr Scand 67: 361–370 6880820 10.1111/j.1600-0447.1983.tb09716.x

